# Distinct gene-set burden patterns underlie common generalized and focal epilepsies

**DOI:** 10.1016/j.ebiom.2021.103588

**Published:** 2021-09-24

**Authors:** Mahmoud Koko, Roland Krause, Thomas Sander, Dheeraj Reddy Bobbili, Michael Nothnagel, Patrick May, Holger Lerche, Epi25 Collaborative

**Affiliations:** a Department of Neurology and Epileptology, Hertie Institute for Clinical Brain Research, University of Tübingen, Otfried-Müller Str. 27, Tübingen 72076, Germany; b Luxembourg Centre for Systems Biomedicine, University of Luxembourg, 6 Avenue du Swing, Belvaux 4367, Luxembourg; c Cologne Center for Genomics, University of Cologne, Faculty of Medicine and Cologne University Hospital, Weyertal 115b, Cologne 50931, Germany; d Cologne Center for Genomics, University of Cologne, Faculty of Mathematics and Natural Sciences and Cologne University Hospital, Weyertal 115b, Cologne 50931, Germany

**Keywords:** Burden analysis, Ultra-rare variants, Gene-sets, Epilepsy, Exome sequencing

## Abstract

**Background:**

Analyses of few gene-sets in epilepsy showed a potential to unravel key disease associations. We set out to investigate the burden of ultra-rare variants (URVs) in a comprehensive range of biologically informed gene-sets presumed to be implicated in epileptogenesis.

**Methods:**

The burden of 12 URV types in 92 gene-sets was compared between cases and controls using whole exome sequencing data from individuals of European descent with developmental and epileptic encephalopathies (DEE, *n* = 1,003), genetic generalized epilepsy (GGE, *n* = 3,064), or non-acquired focal epilepsy (NAFE, *n* = 3,522), collected by the Epi25 Collaborative, compared to 3,962 ancestry-matched controls.

**Findings:**

Missense URVs in highly constrained regions were enriched in neuron-specific and developmental genes, whereas genes not expressed in brain were not affected. GGE featured a higher burden in gene-sets derived from inhibitory vs. excitatory neurons or associated receptors, whereas the opposite was found for NAFE, and DEE featured a burden in both. Top-ranked susceptibility genes from recent genome-wide association studies (GWAS) and gene-sets derived from generalized vs. focal epilepsies revealed specific enrichment patterns of URVs in GGE vs. NAFE.

**Interpretation:**

Missense URVs affecting highly constrained sites differentially impact genes expressed in inhibitory vs. excitatory pathways in generalized vs. focal epilepsies. The excess of URVs in top-ranked GWAS risk-genes suggests a convergence of rare deleterious and common risk-variants in the pathogenesis of generalized and focal epilepsies.

**Funding:**

DFG Research Unit FOR-2715 (Germany), FNR (Luxembourg), NHGRI (US), NHLBI (US), DAAD (Germany).

**Research in context**

**Evidence before this study**

A systematic analysis of specific neuronal gene-sets underlying common generalized vs. focal epilepsies and developmental and epileptic encephalopathies (DEE) has not been performed to date. To evaluate the available evidence, we searched PubMed for articles published before 31.12.2019 using the English search terms “epilepsy AND gene-sets” or “epilepsy AND burden analysis”, identifying three studies with relevant positive findings. The first study examined generalized and focal epilepsies showing a preferential excess of ultra-rare variants in known disease genes in genetic generalized epilepsy (GGE) and non-aquired focal epilepsy (NAFE), and the second investigated generalized epilepsies highlighting the importance of GABA_A_ (inhibitory) receptors in GGE. The third described multiple similarities between rare and common epilepsies in the pattern of rare genetic factors in known disease genes and genes that are most intolerant to genetic variation.

**Added value of this study**

We performed an extensive analysis of biologically informed gene-sets in GGE and NAFE. Leveraging an array of metrics to enrich our analysis for pathogenic variants, we detected a substantial difference in the genetic burden between cases and controls in key neuronal gene-sets, including synaptic and developmental genes. We observed a relatively higher burden in inhibitory vs. excitatory neuronal gene-sets in generalized epilepsy but an increased burden in excitatory vs. inhibitory sets in focal epilepsies. Also, we found an excess of ultra-rare variants in generalized and focal epilepsy cases vs. controls in genes otherwise implicated by genome-wide association studies targeting common variants in the same epilepsy types, suggesting that rare and common variants work in concert to cause these common diseases. These novel results add a wider biological context to the previous findings, improving our understanding of the neuronal processes underlying seizure disorders.

**Implications of all the available evidence**

Ultra-rare genetic variants in individuals with common epilepsies affect shared gene-sets, highlighting the importance of variation-intolerant sites, but also show specific enrichment patterns that suggest a central role for inhibitory and excitatory pathway defects in generalized and focal epilepsies, respectively, and a convergence of genetic risk caused by common and rare variants in both.

Alt-text: Unlabelled box

## 1 Introduction

Dismantling the genetic architecture behind epilepsy is yet to be within reach in many individuals. The role of genetic causality is apparent in the developmental and epileptic encephalopathies (DEEs) [1], [2], [3], sometimes with consequences on precision treatments [4], [5], [6], [7]. In contrast, only few individuals with familial or sporadic genetic generalized epilepsies (GGEs) or non-acquired focal epilepsies (NAFEs) harbour monogenic causative variations [8], [9], [10], [11]. Therefore, methodologies investigating the mutational burden of neurobiologically meaningful gene-sets improve the prospects to dissect the joint effects of multiple genetic factors underlying the complex genetic architecture of these common epilepsy syndromes. Such ‘gene-set analysis’ approaches are likely to provide valuable insights into the role of certain gene-sets and pathways in epilepsy. Recent gene-set burden analyses have shown an enrichment in ultra-rare deleterious and intolerant variants both in common and rare epilepsies in genes associated with dominant epilepsy syndromes, DEE genes, and neuro-developmental disorders (NDDs) with epilepsy genes, emphasizing a shared genetic component [8,11]. Evidence for the enrichment of rare missense variants in genes encoding GABA_A_ receptors and GABAergic pathway genes in GGE pointed to the importance of the inhibitory pathway [9,10]. We used the large-scale dataset collected by the Epi25 Collaborative [10] for a comprehensive, exome-based case-control study to examine the burden of ultra-rare variants (URVs) in a large number of candidate gene-sets for three different epilepsy forms (DEE, GGE, NAFE), aiming to understand the specific roles of deleterious URVs in key pathways implicated in epileptogenesis. Focusing on regional constraint and paralog conservation, we identified relevant and specific gene-set associations in these three epilepsy forms.

## 2 Methods

### 2.1 Study samples

The Epi25 Collaborative collected and generated phenotyping and exome sequencing data from individuals with different subtypes of epilepsy [10]. We analyzed subjects from recruitment years 1 and 2 (*n* = 13,197 before filtering) targeting individuals diagnosed with DEE (*n* = 1,474), GGE (*n =* 4,510), NAFE (*n* = 5,321). The epilepsy classification, phenotyping and consent procedures have been previously described [10,11]. Five control cohorts [10,12] were available for this analysis (*n* = 13,299), including Italian controls from the Epi25 Collaborative (*n* = 300), the Swedish Schizophrenia Study controls (*n* = 6,242), and three Myocardial Infarction Genetics (MIGen) Consortium cohorts: Leicester UK Heart Study (*n* = 1,165), Ottawa Heart Study (*n* = 1,915) and the Italian Atherosclerosis, Thrombosis, and Vascular Biology (ATVB) Study (*n* = 3,677). The ethical approval and consents procedures for the individual cohorts were reported by the Epi25 Collaborative [10]. Subjects investigated by the Epi25 Collaborative provided signed informed consent at the participating centres according to local national ethical requirements and their standards at the time of collection. Approval for data reuse and analysis was obtained from the Epi25 Collaborative (cases) and dbGAP (controls). The data generation process has been previously described [10] (see the Appendix).

### 2.2 Quality control

We considered Non-Finnish European (NFE) individuals diagnosed with DEE, GGE, or NAFE. The ancestry was predicted based on 1000 Genomes data [13] using a Support Vector Machine, removing 1,911 individuals with epilepsy and 146 controls. The quality control procedures [14], [15], [16], [17], [18], [19], [20], [21], [22] aimed to ensure adequate case-control matching and minimize the coverage and call rate differences between cohorts. The final analysis set included 7,589 cases (DEE = 1,003, GGE = 3,064, NAFE = 3,522) and 3,962 matched controls (ATVB = 1,673, Leicester = 1,082, Ottawa = 924, Epi25 Italian = 283). The details are outlined in the supplemental methods (see the Appendix). The use of predominantly male or male-only control cohorts from ATVB and Leicester studies resulted in a misbalanced sample sex ratio (53·6% female cases vs. 19·4% female controls). The effect of this imbalance was addressed in a secondary analysis as will be detailed.

### 2.3 Qualifying variants

The variants were annotated using snpEff [23] v4.3 and Annovar [24] v20191024. We focused on URVs as these have shown a strong burden of deleterious pathogenic variants in multiple studies of epilepsy and other neurological disorders [8,10,11,[25], [26], [27], [28], [29]]. URVs were defined based on their Minor Allele Counts (MACs) in the study dataset (internal allele count/frequency) and their estimated frequency in the general population (external Minor Allele Frequencies, MAFs). Specifically, we examined variants that are: (i) Seen in less than three cases and controls (MAC ≤ 3); (ii) Not seen in DiscovEHR [30] (MAF in DiscovEHR = 0; (iii) Seen at a very low allele frequency in gnomAD [31] r2.1 database (MAF in gnomAD ≤ 2 × 10^–5^). We performed three separate analyses for the three epilepsy phenotypes; Therefore, MACs were calculated independently in each analysis. This was intended to provide a better control for inflation compared to calculating MACs from all cases and controls. Accordingly, the reported variant counts in the control sets may differ slightly between the three analyses. Since our controls overlapped partially with gnomAD r2.1, we did not require complete absence of variants in gnomAD. URVs were categorised further into multiple classes based on their functional consequences and collapsed by gene as qualifying variants (QVs). We considered twelve non-synonymous variant classes including protein-truncating variants (presumed loss-of-function) and multiple groups of missense variants (mix of neutral, loss-, and gain-of-function mechanisms) as well as a (thirteenth) synonymous control classes of variants (presumed neutral). The grouping of missense QVs in multiple (partially overlapping) classes focused on three perspectives: conventional in-silico deleteriousness, constraint and paralog conservation. It was based on multiple predictions, namely, PolyPhen2 [32] (PPh2), Sorting Intolerant From Tolerant [33] (SIFT), Missense Badness Polyphen and Constraint [34] (MPC), Missense Tolerance Ratio [35] (MTR), Constrained Coding Regions [36] (CCR) and para-*Z*-score for paralog conservation [37,38]. The rationale behind the use of these scores is detailed in the supplemental methods (see the Appendix). The analyzed functional classes of variants (Table S6) were: (i) Benign missense variants: as predicted by PPh2 and SIFT. (ii) Damaging missense variants: as predicted by PPh2 and SIFT. (iii) Protein Truncating Variants (PTVs) that included stop-gained, start-lost, frameshift, splice-donor, and splice-acceptor variants. (iv) All functional variants combined: PTVs, in-frame indels, and damaging missense variants. (v) “MPC 1” missense variants: constrained missense with MPC score ≥ 1. (vi) “MPC 2” missense variants: highly constrained missense with MPC score ≥ 2 (enriched for *de novo* variants). (vii) “MTR ClinVar” missense variants: constrained missense with MTR score ≤ 0·825 which is the median for ClinVar variants not denoted as *de novo*. (viii) “MTR De Novo” missense variants: highly constrained missense with MTR score ≤ 0·565 which is the median for ClinVar *de novo* variants. (ix) “CCR 80” missense variants: highly constrained missense variants in regions with CCR score ≥ 80, with MPC score ≥ 1, and MTR score ≤ 0·825. (x) “paralog-non-conserved”: missense variants located in sites not conserved across paralog genes as indicated by a para-*Z*-score ≤ 0. (xi) “paralog-conserved”: missense variants located in sites conserved across paralog genes as indicated by a para-*Z*-score > 0. (xii) “paralog highly conserved”: missense variants in highly conserved sites between paralog genes with para-*Z*-score ≥ 1. (xiii) “Synonymous” variants that served as a control class for inflation.

### 2.4 Gene-sets

92 gene-sets were tested. In addition to exome-wide burden testing (one gene-set of all protein coding genes), we defined additional 91 specific gene-sets as follows: (a) 34 sets based on gene expression patterns in the brain and genic intolerance [39], [40], [41], [42], [43], [44], [45], [46], [47], [48], [49]; (b) 28 functional groups including ion channels [8], GABA_A_ receptors [9], excitatory receptors [9], GABAergic pathway [9], PSD-95 interactors [8], Gene Ontology (GO) gene-sets of GABAergic and glutamatergic synapses [41,42,50], neuronal pathways from Kyoto Encyclopedia of Genes and Genomes [51] (KEGG) and neuronal gene-sets from Reactome [52] database; (c) 14 gene-sets of known disease-related genes including monogenic epilepsy-causing genes [8], [9], [10], epilepsy genome-wide association study (GWAS) top-ranked genes from positional mapping (within a window of 250 kb around significant loci) plus mapping based on chromatin interaction (between gene promoters and the significant locus) [53], co-regulated genes in the brain [54,55]; and (d) 15 non-neuronal gene-sets [51]. The gene-sets are outlined in Table 1 (also see the Appendix).

**Table 1:** **Gene-sets investigated in this study.** The number of gene-sets in each category is given in parenthesis.

**Group of all protein coding genes (1):**		
-all genes annotated by snpEff as protein coding.		
**Groups based on brain expression (34):** Expression in the brain, regional, cellular and sub-cellular expression patterns.		
***Brain-expressed LOF-intolerant genes:*** excluding genes with no expression in the cortex/hippocampus -pLI > 0.995. -pLI 0.9-0.995. -pLI 0.8-0.9. ***Brain-expressed missense-intolerant genes:*** excluding genes with no expression in the cortex/hippocampus -Z-score > 3.09. -Z-score 2.5-3.09. -Z-score 2-2.5.	***Cortical and hippocampal expression level:*** -High, Moderate, Low in the cortex. -High, Moderate, Low in the hippocampus. ***Brain development:*** -Brain development genes (Gene-Ontology group). -Brain developmental genes (extended group). -Early developmental genes. -Late developmental genes. ***Enrichment in the brain:*** -Brain-enriched -Brain-enhanced.	***Cell-type-specific enrichment:*** -Neurons -glial cells -Excitatory neurons -Inhibitory neurons -Astrocytes -Microglia -Oligodendrocytes -Endothelium. ***Neuronal Localization:*** -Axon Initial Segment. -Synaptic (curated group). -Synaptic (extended group). -Synaptic vesicle and active zone. -Pre-synaptic. -Post-synaptic. -Pre-synaptic only. -Post-synaptic only.
**Functional gene-sets (28):** Ion channels, transporters, synaptic cycles, pathways and neurotransmitter cycles.		
***Ion channels, neurotransmitter receptors and related genes:*** -Voltage-gated ion channels. -Voltage-gated cation channels. -Brain-specific voltage-gated ion channels. -GABA_A_ receptors. -GABAergic pathway. -Excitatory receptors. -NMDAR & ARC interactors. -PSD-95 interactors. ***GABAergic/Glutamatergic synapses (GO groups):*** -GABAergic synapse -Glutamatergic synapse -only in GABAergic -only in glutamatergic -shared genes.	***GABAergic/Glutamatergic pathways (KEGG database):*** -GABAergic pathway -Glutamatergic pathway -only in GABAergic -only in glutamatergic -shared genes. ***Additional neuronal pathways (KEGG):*** -Cholinergic pathway. -Dopaminergic pathway. -mTOR pathway. -Synaptic vesicle cycle. Glutamate release, uptake and clearance cycle.	***GABA/glutamate cycles (Reactome database; pooled from multiple groups):*** -GABA release, receptor activation, and clearance - ***Additional neuronal groups (Reactome database):*** -Presynaptic depolarization. -Neurexins and Neuroligins. -Synaptic Adhesion molecules. -Receptor-type Protein Tyrosine Phosphatases.
**Disease-associated and intolerant genes (14):** Genes and gene-sets with known associations with epilepsy and related neurological diseases		
***Monogenic disease-causing genes:*** -Generalized epilepsy genes. -Focal epilepsy genes. -Dominant epilepsy genes -DEE genes. -NDD with epilepsy genes. -FMRP targets. -MGI seizure genes.	***Top-ranking 100 genes in ILAE2 GWAS:*** -Generalized epilepsy GWAS. -Focal epilepsy GWAS. -All epilepsies GWAS. ***Brain co-expression module:*** -Co-expressed module identified in non-diseased post-mortem brain tissues. (enriched for *de novo* variants in DEE).	***Regulatory and co-expression modules in epilepsy:*** -Co-expression network identified in brain tissues of Temporal Lobe Epilepsy patients - Two modules within this network.
**Control groups (15):**		
***Genes not expressed in the brain:*** -RNA not detected in cortex, in hippocampus, or all GTEx regions. -Protein is depleted in the brain.	***KEGG metabolic pathways:*** -Type II Diabetes. -Carbohydrate Absorption & Digestion. -Protein Absorption & Digestion. -Fat Absorption & Digestion.	***KEGG cancer pathways:*** - CA Breast, CA Lung, CA Colon, CA Prostate, Renal Cell Ca, CA Pancreas, Hepatocellular Ca.

### 2.5 Gene-set burden analysis

We examined the burden of QVs in thirteen variant classes (Table S6) for 92 gene-sets in three epilepsy phenotypes (DEE, GGE, and NAFE) against a set of matched controls. Gene-set burden testing was done using logistic regression by regressing the case-control status on the individual QVs counts. In each sample, URVs that matched the specific analysis criteria were collapsed by gene into QVs (each sample was a assigned a status indicator: 1 for the presence of a QV or 0 for its absence) and these QVs were aggregated (summed per sample) across a target gene-set to get a burden score (assuming equal weights and direction of effects) which was used as a predictor in a binomial model while adjusting for additional covariates (sex, top ten principal components, exome-wide variant count, and exome wide singletons count) using *glm*() function from *stats* package [56]. Likelihood ratio test (LRT) from *lmtest* package [57] was used to compare a model with QVs burden and covariates as predictors against a null model (covariates only). Log-odds from LRT and their respective 95% confidence intervals and *p* values are presented here as a measure of enrichment in tested gene-sets. We employed a Benjamini-Hochberg false discovery rate (FDR) multiple testing adjustment for *p* values that accounted for 3,312 tests (92 gene-sets x 3 epilepsy phenotypes x 12 non-synonymous variant classes, excluding synonymous variants used as a control class) as implemented in *p.adjust*() function from *stats* package [56]. The cut-off for substantial enrichment was defined as FDR-adjusted *p* value < 0·05. For simplicity, *p* values (FDR-adjusted except for synonymous variants) are indicated throughout the presented plots using stars as follows: no star > 0·05, * < 0·05, ** < 0·005, *** < 0·0005, **** < 0·00005. To estimate the extent of bias that might have been introduced by the imbalance in male-to-female ratios between cases and controls, we performed a secondary analysis excluding chromosome *X* genes (Table S11). Also, to ensure adequate control for any bias introduced by differences in capture kits, we performed another secondary analysis between two groups of control samples (Leicester study controls vs. Ottawa and ATVB controls) representing two main enrichment kits (1,100 samples enriched using Illumina ICE kits vs. 2,789 samples enriched using Agilent SureSelect kits). To ensure that the latter analysis would have sufficient power, it was coupled with an analysis of randomly selected individuals with GGE (*n* = 1,100) vs. controls (*n* = 2,789) using the CCR 80 class of variants. We did 500 permutations, taking the average odds, 2.5^th^/97.5^th^ centiles of odds and average *p* values as an outcome. Lastly, to explore the extent of the observed differences between GGEs and NAFEs, we performed another limited secondary analysis comparing the CCR80 class of variants directly between individuals with GGE and NAFE. The statistical analysis was performed in R [56] v3.3.3. The analysis approach is outlined in Fig. S1 (see the Appendix). Details of the analysis methods, tables of tested gene-sets (Table S7) and genes in each set (Table S8) are provided in the Appendix.

### 2.6 Role of the funding source

The funding agencies had no role in study design; in the collection, analysis, and interpretation of data; and in the writing and the decision to submit the paper for publication. The authors did not receive payments from companies or other agencies to write this article, had full access to the data in the study and accept final responsibility to submit for publication.

## 3 Results

### 3.1 URVs excess in brain-expressed genes

**Fig. 1:**
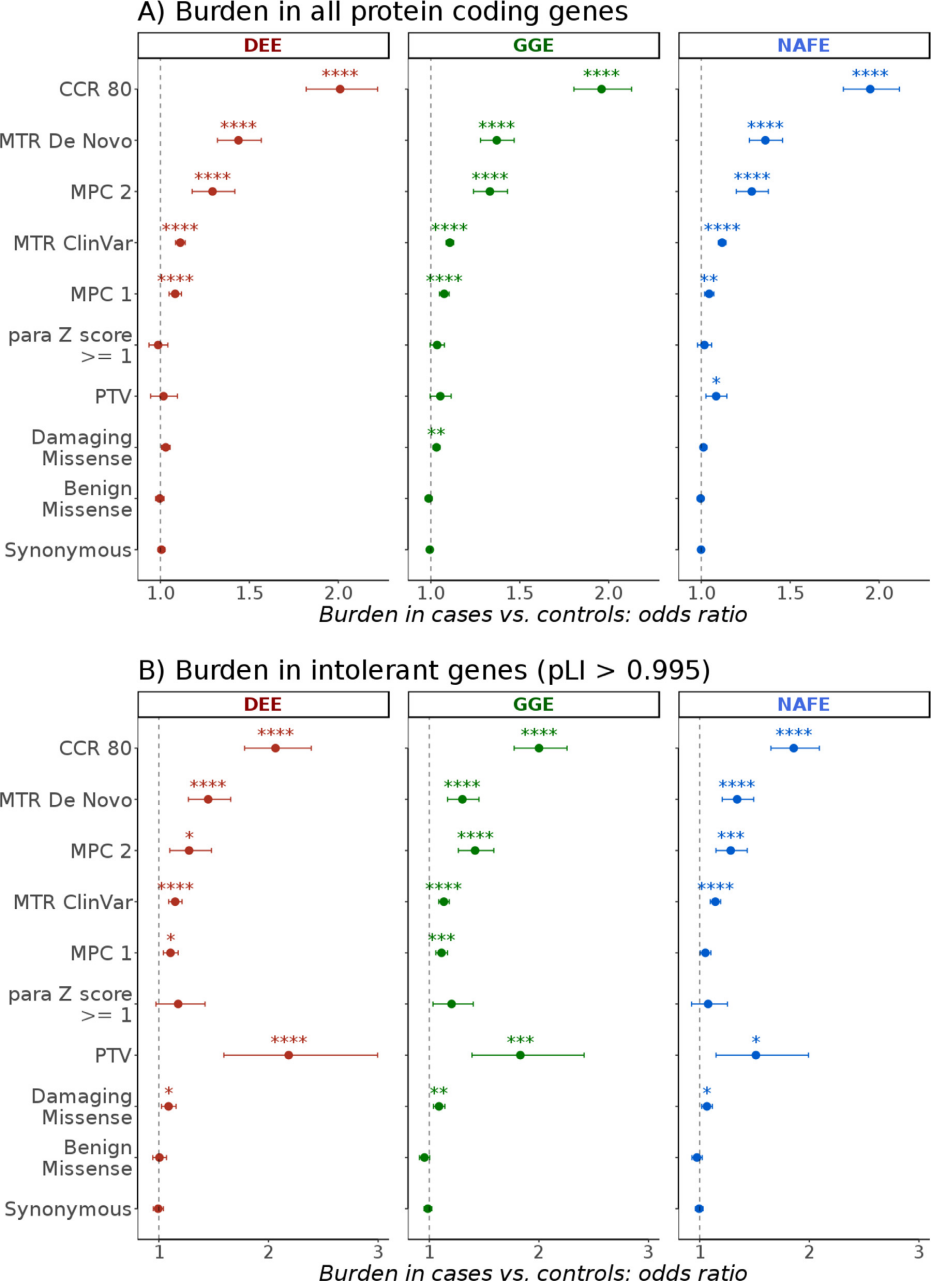
**Exome-wide burden of ultra-rare variants in the epilepsies.** The burden in developmental and epileptic encephalopathies (DEE), genetic generalized epilepsies (GGE) and non-acquired focal epilepsies (NAFE) in **(A)** 19,402 protein coding genes and **(B)** 1,743 genes with probability of loss-of-function intolerance (pLI) score > 0·995 is shown in multiple classes of variants (y-axis; see methods) as odds ratio (*x*-axis) from Likelihood Ratio Test (bars indicate 95% confidence intervals). False-Discovery-Rate-adjusted *p* values (synonymous variants analysis *p* values were not adjusted) are indicated with stars as follows: no star > 0·05, * < 0·05, ** < 0·005, *** < 0·0005, **** < 0·00005.

First, we investigated the burden of URVs across all protein coding genes following the analysis approach outlined in Fig. S1. This revealed a clear enrichment in constrained missense variants that was maximum in consensus constrained coding regions predicted by Missense-badness Polyphen and Constraint (MPC), Missense Tolerance Ratio (MTR) and Consensus Coding Regions (CCR) scores (Fig. 1). The combination of the three metrics identifies highly deleterious variants in functionally critical genic regions (see methods). In this particular analysis in all three phenotypes, about half of the cases, in contrast to roughly one-fourth of controls, harboured one or more QVs in highly constrained regions (Fig. S11). A previous similar analysis of this [10] and related [11] datasets examined loss-of-function intolerant genes and demonstrated an increased burden in ultra-rare constrained as well as protein truncating variants (PTVs). Here, the examination of brain-expressed intolerant genes showed, similarly, a marked enrichment in PTVs in addition to a burden in highly constrained missense variants that is comparable to what is seen exome-wide (Figs. 1 and S12).

**Fig. 2:**
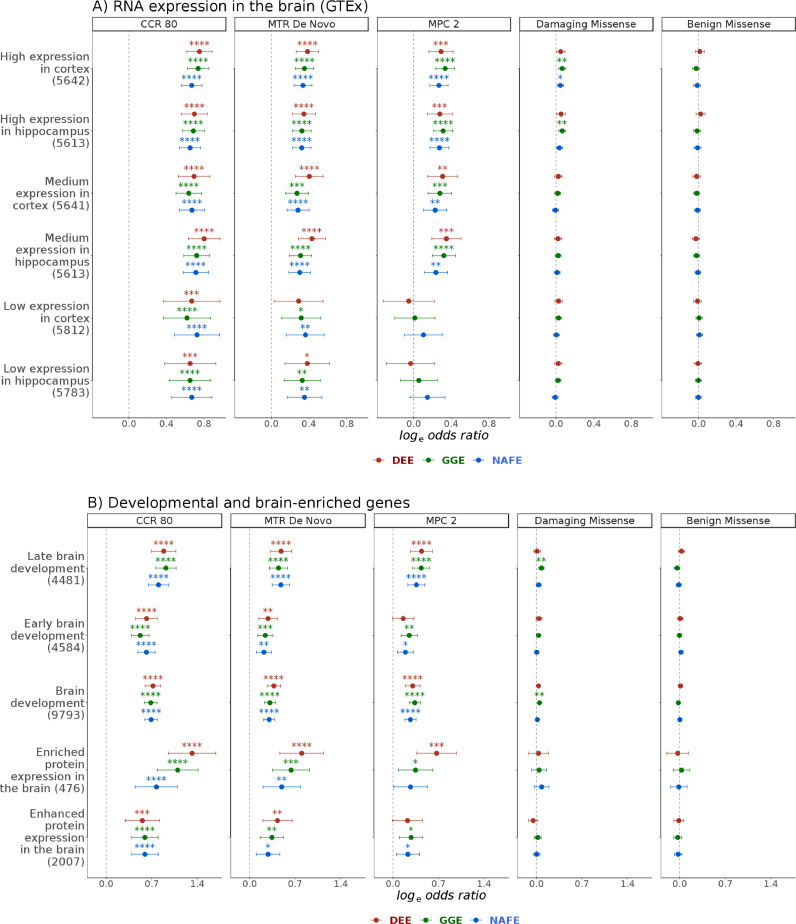
**Burden of ultra-rare missense variants in brain expressed and developmental genes.** The burden of benign or damaging missense variants and missense variants in highly constrained sites in developmental and epileptic encephalopathies (DEE), genetic generalized epilepsies (GGE) and non-acquired focal epilepsies (NAFE) is shown in gene-sets based on levels of RNA/protein expression in the cortex and hippocampus **(A)** or enrichment in adult or developing brain **(B)**. Gene-sets are shown on the y-axis (number of genes in parenthesis). Log odds ratio (Likelihood Ratio Test) are shown on the x-axis (error bars indicate 95% confidence intervals). The variant classes are shown in vertical panels. False-Discovery-Rate-adjusted *p* values are indicated with stars as follows: no star > 0·05, * < 0·05, ** < 0·005, *** < 0·0005, **** < 0·00005. High, medium and low expression categorization was based on expression levels in Gene Tissue Expression ​Project portal (GTEx). Brain-enriched (with more than four-fold expression in the brain compared to other tissues) and brain-enhanced genes (higher but less than four-fold expression) were obtained from the Human Protein Atlas.

When we examined protein coding genes grouped by their relative brain expression, damaging missense variants were only substantially enriched in genes highly expressed in the cortex or hippocampus, whereas those expressed at medium or low levels only showed an enrichment for the most constrained missense variants (Fig. 2). Genes with depleted expression in the brain did not show a substantial enrichment for any variant type (Fig. S18). Genes showing a higher expression in the adult brain compared to other tissues (brain-enriched & brain-enhanced) were also preferentially enriched, as well as genes associated with brain development. Genes related to late rather than early development showed a slightly higher enrichment in all three phenotypic groups (Fig. 2).

**Fig. 3:**
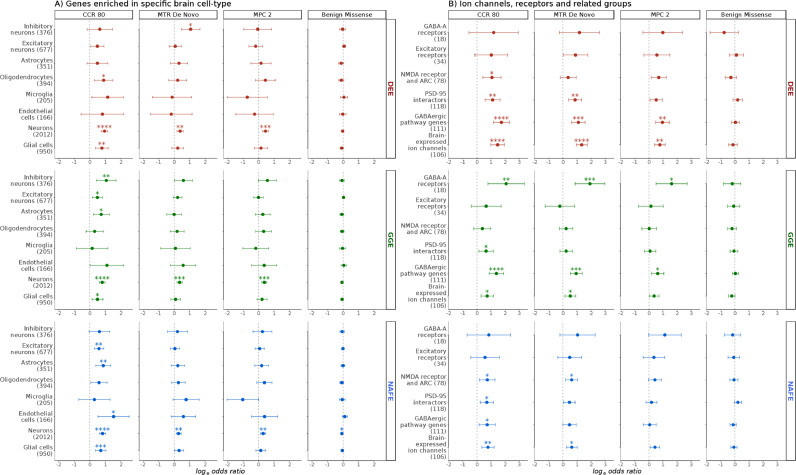
**Burden in neuronal and glial cells, ion channels, receptors and related interactors.** The burden in developmental and epileptic encephalopathies (DEE), genetic generalized epilepsies (GGE) and non-acquired focal epilepsies (NAFE) is shown on the x-axis (log-odds from Likelihood Ratio Test; error bars indicate 95% confidence intervals). Gene-sets are shown on the y-axis (number of genes in parenthesis). The variant classes are shown in vertical panels. False-Discovery-Rate-adjusted *p* values are indicated with stars as follows: no star > 0·05, * < 0·05, ** < 0·005, *** < 0·0005, **** < 0·00005. **(A)** Burden in genes enriched in specific brain cells including neuron- or glia-enriched genes and their subtypes. **(B)** Burden in key biologically informed neuronal gene-sets with known or suspected relation to epilepsy. NMDA: N-Methyl-Dextro-Aspartate. ARC: neuronal activity-regulated cytoskeleton-associated protein (interactors). PSD-95: Post-Synaptic-Density protein 95 (interactors).

Focusing further on cell-type specific expression, neuron-specific genes were preferentially affected compared to those enriched in glial cells, particularly in GGE (Fig. 3). To obtain further insights into the nature of this neuronal enrichment, we used sets of genes representing paralogs of mouse genes found to be enriched in excitatory or inhibitory neurons (see the Appendix). Interestingly, genes preferentially expressed in inhibitory neurons showed an increased burden only in GGE, whereas those preferentially expressed in excitatory neurons showed a more prominent signal in NAFE. Since well-established epilepsy genes, like ion channels and receptors, show differential distributions in different neuronal compartments [58,59], we examined further sets of genes based on subcellular localization. We found that pre- and postsynaptic genes were enriched with variants in cases vs. controls, as well as a very small set of 17 genes located in axon initial segments (most prominent in DEE) (Fig. S16).

### 3.2 Burden of URVs in ion channel, neurotransmitter receptor encoding and related genes

Next, we examined functional gene-sets that could, more specifically, underlie the observed enrichment in neuronal and synaptic genes. Ion channels, neurotransmitter receptors and transporters are widely implicated in epilepsy, especially in monogenic and familial forms, displaying considerable phenotypic heterogeneity and presenting as mild or severe epilepsies [60], [61], [62]. Variants in GABA_A_ receptors were enriched in GGE but not in DEE or NAFE while those in gene-sets representing genes encoding N-Methyl-D-Aspartate receptor and Activity-Regulated Cytoskeleton protein [8] (NMDAR-ARC) interactors were enriched in NAFE and DEE. A comprehensive gene-set for the GABAergic pathway genes [9] showed a prominent signal in GGE and DEE, and less in NAFE. In contrast, a gene-set representing PSD-95 interactors showed comparable enrichment in NAFE and GGE (Fig. 3). Brain-expressed ion channels were found to be enriched for highly constrained missense variants (CCR 80 class of variants) in common as well as rare epilepsies (Fig. 3).

### 3.3 Patterns of burden in gene-sets representing inhibitory vs. excitatory signalling

**Fig. 4:**
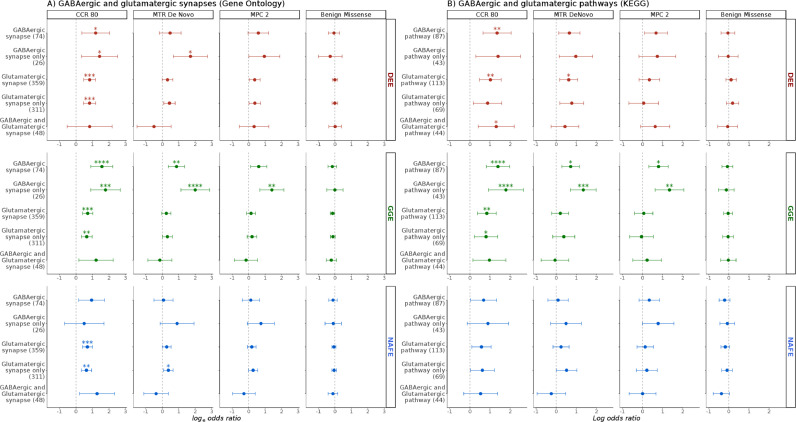
**Enrichment in major neuronal synapses and pathways.** Panels show comparison of enrichment patterns in developmental and epileptic encephalopathies (DEE), genetic generalized epilepsies (GGE) and non-acquired focal epilepsies (NAFE) in GABAergic and glutamatergic synapses and pathway genes based on **(A)** Gene-Ontology (GO) and **(B)** Kyoto Encyclopaedia for Genes and Genomes (KEGG). The burden is shown on the x-axis (log-odds from Likelihood Ratio Test; error bars indicate 95% confidence intervals). Gene-sets are shown on the y-axis (number of genes in parenthesis). The variant classes are shown in vertical panels. False-Discovery-Rate-adjusted *p* values are indicated with stars as follows: no star > 0·05, * < 0·05, ** < 0·005, *** < 0·0005, **** < 0·00005. Complete groups, genes specific to one of the two synapses/pathways as well as their intersection were tested.

We then compared the patterns of URVs burden in genes involved in the GABAergic (main inhibitory) pathway and synapse against those in the glutamatergic (main excitatory) pathway and synapse in the brain, by examining their unique and overlapping genes based on KEGG pathways [51] or GO synaptic gene-sets [41] and sets of specific receptors (Fig. 4). GGE showed a higher burden in GABAergic vs. glutamatergic synapse (GO) and pathway (KEGG) genes, in genes encoding GABA_A_ receptors vs. excitatory receptors/NMDAR-ARC interactors, and in GABAergic pathway genes (comprehensive gene-set) vs. genes encoding PSD-95 interactors, thus matching the higher burden in genes representing inhibitory vs. excitatory neuronal signalling. The CCR 80 analysis of GO gene-sets in NAFE showed a higher burden in glutamatergic vs. GABAergic synapse genes, akin to the pattern seen in genes enriched in excitatory vs. inhibitory neurons. The analysis of KEGG glutamatergic vs. GABAergic pathway genes did not confirm this finding (Fig. 4). It is notable that the overlap between GO synapse and KEGG pathway gene-sets is minimal, and the size of GO and KEGG gene-sets was comparable in GABAergic but discordant in glutamatergic genes (Fig. S21).

Altogether, these comparisons of the burden in missense variants in highly constrained sites between GGE and NAFE (Figs. 3 and 4) suggest the following patterns: (i) brain-expressed ion channels, genes enriched in excitatory neurons, enriched in astrocytes, PSD-95 interactors, GABAergic and glutamatergic synapse/pathway genes show an increased burden in cases vs. controls both in GGE & NAFE; (ii) in GGE, this enrichment is coupled with a stronger enrichment in inhibitory neuronal genes, in genes coding for GABA_A_ receptors and in GABAergic synapse-specific genes (higher burden in inhibitory vs. excitatory gene-sets); and (iii) in NAFE, this is accompanied by an absence of enrichment in the later gene-sets and increased burden in the NMDAR-ARC interactors gene-set (higher burden in excitatory vs. inhibitory gene-sets). A direct comparison of GGEs vs. NAFEs supported the observation of a substantially higher burden of highly constrained variants (CCR 80 class of missense variants) in GABAergic pathway genes in GGEs (Fig. S15).

### 3.4 Burden in gene-sets of known epilepsy-related genes

**Fig. 5:**
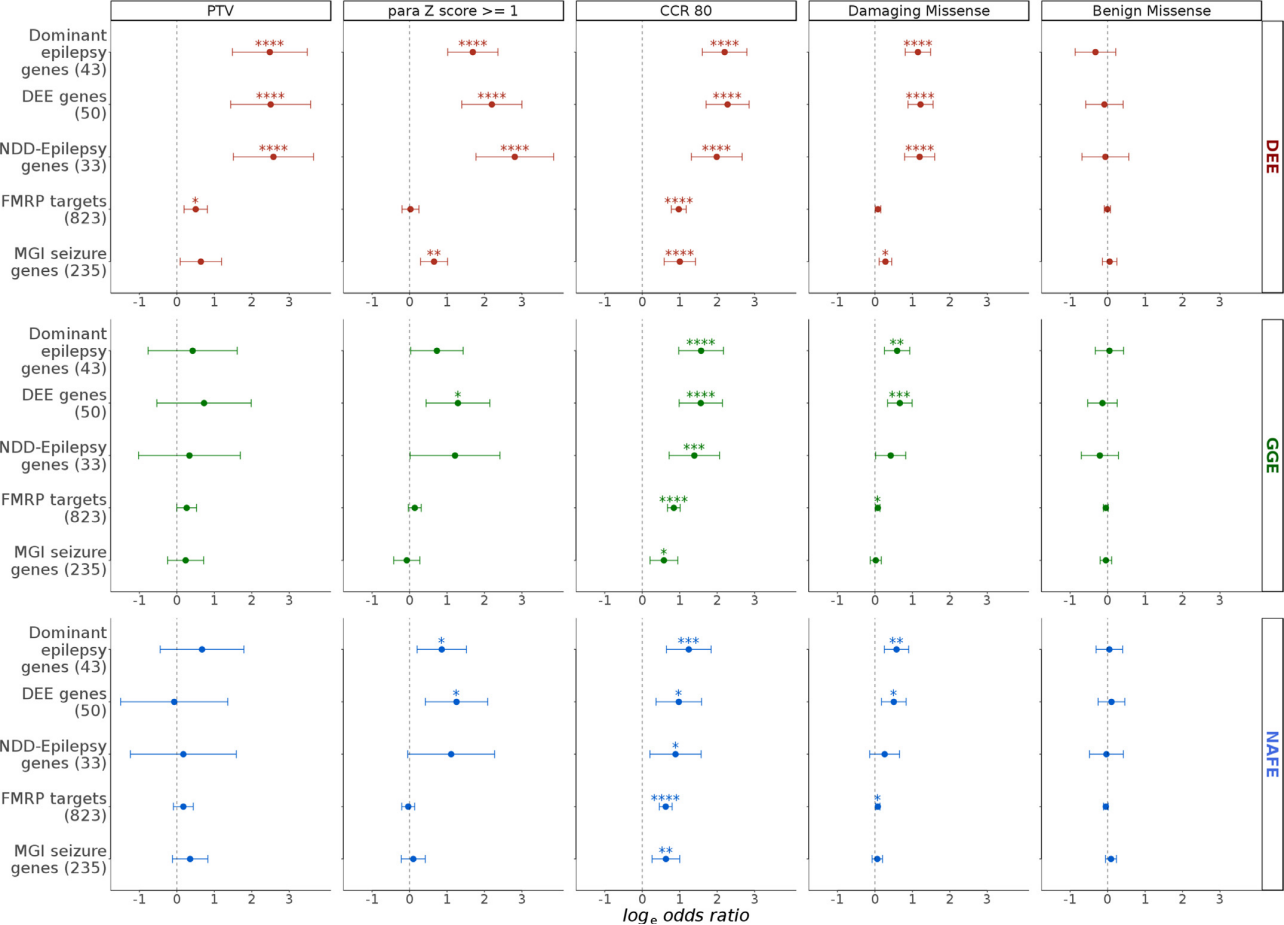
**Burden of ultra-rare variants in groups of epilepsy-related known disease genes.** The burden in five gene-sets (y-axis; number of genes in parenthesis) in developmental and epileptic encephalopathies (DEE), genetic generalized epilepsies (GGE) and non-acquired focal epilepsies (NAFE) (horizontal panel) in selected variant classes (vertical panels) is shown on the x-axis (log odd ratios from Likelihood Ratio Test; error bars indicate 95% confidence intervals). False-Discovery-Rate-adjusted *p* values are indicated with stars as follows: no star > 0·05, * < 0·05, ** < 0·005, *** < 0·0005, **** < 0·00005. NDD-Epilepsy: neurodevelopmental disorders with epilepsy. FMPR: Fragile-*X* Mental Retardation Protein. MGI: Mouse Genome Informatics database.

The previous Epi25 Collaborative analyses [10,11] demonstrated a high burden of missense variants in constrained (intolerant) sites in DEE, GGE, and NAFE, seen in dominant epilepsy genes, DEE genes, and NDD-Epilepsy genes. We observed similar enrichment patterns (Fig. 5) in MPC 2 and MTR De Novo classes of variants (enriched for *de novo* mutations). Limiting the analysis to highly constrained genic regions (CCR 80 class of variants) resulted in a marked increase in URVs burden, as was the trend in all the tested gene-sets so far. Testing these sets also unravelled strong enrichment in PTVs and missense variants in paralog-conserved sites. PTVs and missense variants in paralog-conserved sites did not show substantial enrichment in exome-wide analysis and most of other expression-based, localization-based or pathway-based gene-sets. However, we saw a modest increase in PTV burden in highly intolerant genes with probability of Loss-of-function Intolerance (pLI) > 0·995 in all epilepsies (Fig. 1). The choice of the pLI score cut-off was based on the outcomes of a previous analysis [10] which demonstrated that the burden in PTVs in genes with pLI > 0·9 is driven primarily by genes with pLI > 0·995 rather 0·9–0·995, a pattern that we were able to reproduce (Fig. S12). In a gene-set of known DEE genes, where highly intolerant genes are rather prevalent, we saw a prominent enrichment in PTVs burden in DEE. Also, there was an increased burden in missense variants in paralog-conserved sites in sets of epilepsy-related disease genes (DEE genes, dominant Epilepsy genes, NDD-Epilepsy genes). This burden was very strong in DEE but not as remarkable in GGE and NAFE (Fig. 5).

### 3.5 Enrichment in top GWAS hits captures divergence between common epilepsies

**Fig. 6:**
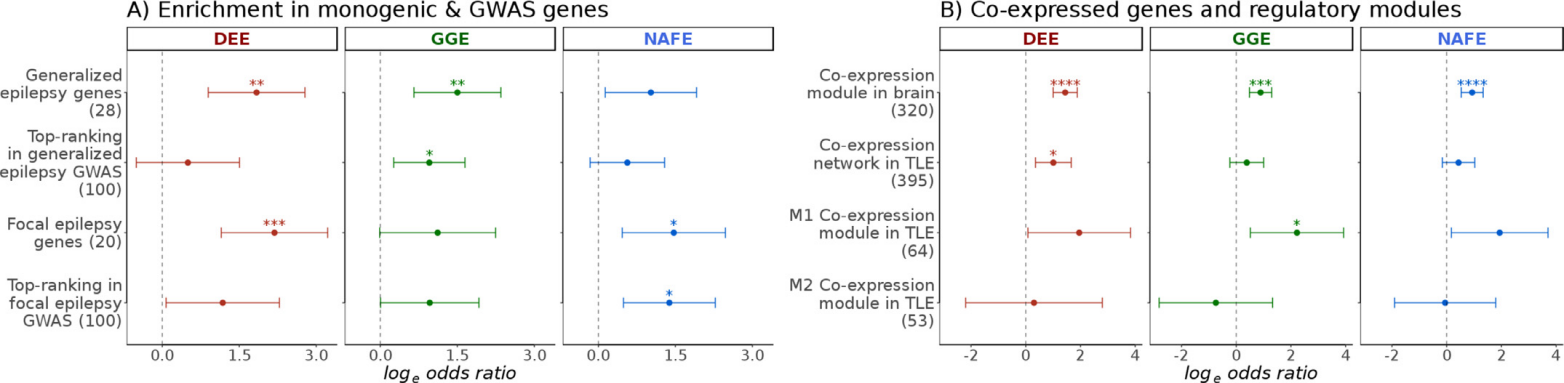
**Risk elements in GWAS top-ranked genes and co-expression modules.** The burden of missense variants in highly constrained sites (log-odds on the x-axis; error bars indicate 95% confidence intervals) in developmental and epileptic encephalopathies (DEE), genetic generalized epilepsies (GGE) and non-acquired focal epilepsies (NAFE) is shown in gene-sets (y-axis; number of genes in parenthesis) representing (**A**) Generalized or Focal epilepsy (presumed monogenic) genes as well as top-ranked 100 genes from GWAS of generalized and focal epilepsies, and (**B**) co-expressed genes identified in post-mortem brain tissues of healthy individuals (module of 320 genes) or in brain tissues from TLE patients (network of 395 genes) as well as two sub-modules of this network (M1 and M2). False-Discovery-Rate-adjusted *p* values are indicated with stars as follows: no star > 0·05, * < 0·05, ** < 0 ·005, *** < 0·0005, **** < 0·00005.

Recent efforts from the International League Against Epilepsy (ILAE) consortium on complex epilepsies identified multiple associations in a large GWAS of common epilepsies [53]. To examine the hypothesis that genes located near the top GWAS hits are also affected by rare variants, we tested the enrichment in sets of the 100 top-ranked genes derived from the ILAE GWAS in generalized, focal, and all epilepsies. Interestingly, when limiting the analysis to Consensus Coding Regions (CCR 80 class of variants), top-ranked genes derived from the GWAS of either generalized or focal epilepsies were preferentially enriched for rare variants in the respective phenotypic groups of GGE and NAFE (Fig. 6). Although the observed enrichment was rather subtle, this result was corroborated by a similar pattern in two, rather small, sets of known epilepsy genes that are predominantly associated with either generalized or focal epilepsy [9].

### 3.6 Brain- and epilepsy-related co-expression modules

We also aimed to touch upon the role of brain co-expression modules identified in post-mortem brain tissues from healthy individuals [55] and to contrast these to the networks and modules identified in brain tissue derived from epilepsy patients [54]. A brain expression module was found to be substantially enriched for rare deleterious variants in an independent cohort of DEE [55]. A link to common epilepsy phenotypes was also inferred, but a burden in URVs was not examined so far. This module showed a non-specific enrichment in all three epilepsy subtypes with highest odds in DEE. It is noteworthy that this module overlaps largely with known epilepsy genes (Fig. S22). In resected hippocampi of individuals with temporal lobe epilepsy (TLE), Johnson and colleagues identified two co-expression modules within a gene-regulatory transcriptional network [54]. A subtle enrichment was seen in these modules in DEE and GGE, but not NAFE (Fig. 6).

### 3.7 Additional neuronal and non-neuronal pathways

Other neuronal gene-sets were enriched in our analysis. Genes encoding neurexins and neuroligins, important elements of pre- and post-synaptic interaction promoting adhesion between dendrites and axons [63], were enriched in DEE (Fig. S17). Also, the synaptic vesicle cycle pathway (KEGG) showed a prominent signal in both DEE and GGE. We also examined the burden in the mTOR pathway (KEGG), hypothesizing that it could have potential relevance to focal epilepsies, but did not detect a substantial enrichment (Fig. S17). Interestingly, NAFE analysis displayed a burden in endothelial and astrocyte-specific genes in highly constrained genic regions (Fig. 3). Detailed results from all tested classes including the counts of genes with observed QVs, variant counts in cases and controls, logistic regression odds of the individual QVs burden in cases vs. controls and related *p* values are provided in Table S9.

### 3.8 Specificity of the observed enrichment patterns

Examination of control classes and control gene-sets that are not expected to show an enrichment supported the validity of our analysis. Four sets of genes not expressed in the brain that were tested (high confidence genes with depleted RNA and protein expression in the brain, genes with no RNA detected in the cortex, the hippocampus or any brain tissue) were not substantially enriched in most of the tested variant classes (Fig. S18). Also, we examined eleven metabolic and cancer pathways (KEGG) to have additional insights into the specificity of the observed signals to neuronal processes and genes. Among 540 tests targeting functional variants in these non-neuronal gene-sets (3 epilepsy subtypes, 15 sets representing genes not expressed in the brain, KEGG metabolic and cancer pathways, 12 non-synonymous functional classes of variants), 18 tests (3·3%) had an FDR-adjusted *p* values < 0·05. At least for some of those, the enrichment could be explained by an overlap with genes known to play a role in epilepsy. For instance, genes forming the Type II Diabetes KEGG pathway are substantially enriched in DEE (FDR-adjusted *p* values of 0·007 for MTR DeNovo and 0·01 for CCR 80 class of variants). This pathway contains two genes that are known to cause DEE, namely, *CACNA1A* [64] and *CACNA1E* [65]. The enrichment was no longer prominent (*p* values > 0·05) after the removal of these two genes (Fig. S23).

### 3.9 Bias and inflation in gene-set burden testing

The analysis for synonymous variants did not show more substantial enrichment than expected by chance, indicating sufficient control for inflation, particularly in exome-wide models and gene-sets with large number of genes. In this control analysis (synonymous variants), few tests showed *p* values < 0·05 (15 out of 276 tests of 92 gene-sets and 3 phenotypes: 5·4%). The analysis for benign missense variants, another class that is not expected to show an increased burden in cases vs. controls [10], did not show substantial enrichment as well. Nine out of 276 tests for benign missense variants (3·2%) showed *p* values < 0·05 (only 2 with FDR-adjusted *p* values < 0·05). Possible alternative explanations for such subtle signals include residual population stratification, differences in exome capture not adjusted by covariates and the presence of synonymous variants with functional consequences [66]. However, these proportions are close to the limit expected by chance under a true null hypothesis (5% with α = 0·05). A potential source of bias in our burden testing was the imbalance in male-to-female ratios between cases and controls (Table S4). We provide results from a secondary analysis that excluded all genes located on chromosome X, which shows that any bias not captured by the inclusion of sample sex as a covariate is likely marginal (Table S11). To exclude any major residual stratification resulting from the use of different enrichment kits, we additionally performed a controls-only analysis in which we compared control samples enriched with Illumina ICE capture kits (from Leicester study) to controls enriched using Agilent SureSelect kits (ATVB study and Ottawa study). This analysis reflected a good control for any potential bias introduced by different exome capture systems and also demonstrated that the mixing of controls included (Leicester and Ottawa) or not included (ATVB) in gnomAD is unlikely to have affected our main outcomes (Table S11).

## 4 Discussion

By analyzing the sequencing data of 11,551 unrelated European individuals (1,003 individuals with DEE, 3,064 individuals with GGE, and 3,522 individuals with NAFE vs. 3,962 controls), we show an increased burden in ultra-rare missense variants in highly constrained sites in epilepsy cases compared to controls, not only in intolerant and known epilepsy-related genes, as previously shown [10,11], but also exome-wide in all protein coding genes. Similar to the observations made in several other phenotypes, the burden in PTVs was most prominent in known disease genes and brain-expressed loss-of-function intolerant genes [31,39,67]. Consistent with their enrichment in neurodevelopmental disorders [37], the burden in missense variants in paralog-conserved sites was prominent in DEEs. The lower burden of these variants in GGEs and NAFEs may reflect a true disparity between rare and common epilepsies. The presented results are also consistent with previous analyses of missense variants in a small number of gene-sets examined in similar cohorts [8], [9], [10], [11].

The systematic analysis of additional gene-sets and a wider variety of classes of variants revealed interesting findings about the neurobiology of distinct types of epilepsy. Although associated with higher odds ratios of an epilepsy phenotype, enriched variants are not deterministic on their own, since about one-fourth of the controls also carry qualifying variants in the CCR 80 analysis (Fig. S11). As such, the phenotype is determined by a constellation of other factors, possibly including the severity of variants [11], patterns of multiple variations, oligogenic contribution from rare variants [68], and polygenic risk from common variants [69]. Developmental genes were key drivers in all epilepsies suggesting that the impairment of developmental processes is not limited to DEEs with marked developmental deficits [70,71]. The enrichment in synaptic genes is another shared feature between the epilepsies that has also been observed in neurodevelopmental disorders with epilepsy [72,73], schizophrenia [25], and autism [74]. This highlights a shared genetic architecture not only between epilepsy subtypes but also with other related neurological disorders, as has been shown previously for common variants [75].

Despite the common genetic and phenotypic features, DEEs, GGEs and NAFEs represent well-recognized phenotypic clusters with defined electro-encephalographic and clinical characteristics. Given the phenotypic severity of DEEs, the prevalence of *de novo* variants and monogenic cases in DEE (those with pathogenic and likely pathogenic variants in known monogenic genes), and the description of phenotypic spectra for genes involved in DEE that also span the milder GGE or NAFE, the distinction between severe and mild epilepsies could be attributed, at least to some extent, to the severity of the genetic defects, their functional effects or their localization within certain channel regions [11,62,[76], [77], [78], [79]]. The distinction between GGE and NAFE, however, is probably functional, at least in part, as suggested by previous work demonstrating the centrality of GABAergic genes in generalized epilepsies [9,10]. Also, it is well recognized that few genes present with focal epilepsy and are not linked generalized epilepsy syndromes [80]. Here, phenotype-specific patterns in gene-sets representing neuronal inhibitory vs. excitatory signalling were observed in comparisons of GGE and NAFE.

Additional disparities in key gene-sets (genes implicated in monogenic generalized & focal epilepsy, the 100 top-ranked genes associated with GWAS hits in generalized & focal epilepsy) point to a possible genetic-functional divergence, so that a common background of shared risk seems to be overlaid by specific risk entities. The enrichment of rare variants in GWAS genes also supports the convergence of ultra-rare and common variants in conferring epilepsy risk, in concordance with the observed enrichment of epilepsy GWAS hits for monogenic epilepsy genes [53]. According to our findings, a link between common and rare variants is likely to be also relevant for the phenotypic heterogeneity observed in seizure disorders. Notably, polygenic risk scores also pointed out the specificity of the risk profiles in common epilepsies [69]. Based on previous findings of an increased URV burden in DEEs [55] and the current findings in GGEs and NAFEs, it is also conceivable that differentially expressed genes in individuals with epilepsy, representing closely orchestrated networks with possible functional correlations, would highlight modules in which altered transcription, URVs, or both contribute to cause both rare and common epilepsies.

The associations presented in this work should be interpreted with the caveats of gene-set testing in mind [81]. Pathways and molecular processes are not consistently defined in different resources (Fig. S21). These differences may explain the discrepancies in enrichment patterns in the same pathway. We examined multiple overlapping gene-sets from different sources to corroborate the findings that underscore a genuine biological relevance. Our analysis has additional limitations which we aimed to overcome using stringent analysis and quality control strategies. The limited use of about half of the controls from the primary analysis affected the overall power. Nevertheless, we were able to reproduce most of the major signals from gene-sets with large effect sizes, the latter thereby acting as positive controls. Multiple secondary analyses suggested that the impalpable of male-to-female ratios in our case and control sets and the use of sequencing data from ExAC [82], gnomAD [31] or DiscovEHR [30] to develop, train or validate *in-silico* algorithms used for estimating constraint [34], [35], [36] do not seem to have introduced a substantial bias (Table S11). The overlap between the controls used in this study and gnomAD controls (Table S2) created some challenges in defining URVs. For population frequency filtering, we allowed around five alleles in gnomAD (allele frequency of 2 × 10^−5^) to retain URVs from our control that are also seen in gnomAD while still filtering common variants and prevalent sequencing artifacts.

In conclusion, missense URVs affecting constrained sites in brain-expressed genes show distinct signatures in epilepsy. Enrichment patterns of URVs-affected genes suggest a preferential involvement of inhibitory genes in GGE and excitatory genes in focal epilepsies. Genes implicated by common GWAS variants may also be disrupted by URVs in various epilepsy phenotypes, suggesting a convergence of rare disruptive variants, and common variants in the pathogenesis of epilepsy.

**Online appendix:** Supplementary materials including supplemental methods, Tables S1–S6, Figs. S1–S20, and affiliations of the Epi25 Collaborative members accompany the online version of this article. Additional large supplemental Tables S7–S12 are available on Mendeley Data.

## Contributors

HL, PM, MK conceived the study. HL, PM, RK, and MK contributed to the study design and/or data acquisition. Authors from the Epi25 Collaborative recruited and phenotyped patients and/or contributed to data generation as indicated in the supplemental author information (see the Appendix). MK and PM performed the analysis and verified the underlying data with input from HL, RK, TS, DRB and MN. MK, PM, and HL interpreted the results and wrote the manuscript with input from TS. All authors reviewed the manuscript for intellectual content and approved the final version of the manuscript. PM and HL contributed equally as PIs.

## Epi25 collaborative

Yen-Chen, Anne, Feng. Daniel, P, Howrigan. Liam, E, Abbott. Katherine, Tashman. Felecia, Cerrato. Tarjinder, Singh. Henrike, Heyne. Andrea, Byrnes. Claire, Churchhouse. Nick, Watts. Matthew, Solomonson. Dennis, Lal. Erin, L, Heinzen. Ryan, S, Dhindsa. Kate, E, Stanley. Gianpiero, L, Cavalleri. Hakon, Hakonarson. Ingo, Helbig. Roland, Krause. Patrick, May. Sarah, Weckhuysen. Slavé, Petrovski. Sitharthan, Kamalakaran. Sanjay, M, Sisodiya. Patrick, Cossette. Chris, Cotsapas. Peter, De Jonghe. Tracy, Dixon-Salazar. Renzo, Guerrini. Patrick, Kwan. Anthony, G, Marson. Randy, Stewart. Chantal, Depondt. Dennis, J, Dlugos. Ingrid, E, Scheffer. Pasquale, Striano. Catharine, Freyer. Kevin, McKenna. Brigid, M, Regan. Susannah, T, Bellows. Costin, Leu. Caitlin, A, Bennett. Esther, M C, Johns. Alexandra, Macdonald. Hannah, Shilling. Rosemary, Burgess. Dorien, Weckhuysen. Melanie, Bahlo. Terence, J, O'Brien. Marian, Todaro. Hannah, Stamberger. Danielle, M, Andrade. Tara, R, Sadoway. Kelly, Mo. Heinz, Krestel. Sabina, Gallati. Savvas, S, Papacostas. Ioanna, Kousiappa. George, A, Tanteles. Katalin, Štěrbová. Markéta, Vlčková. Lucie, Sedláčková. Petra, Laššuthová. Karl, Martin, Klein. Felix, Rosenow. Philipp, S, Reif. Susanne, Knake. Wolfram, S, Kunz. Gábor, Zsurka. Christian, E, Elger. Jürgen, Bauer. Michael, Rademacher. Manuela, Pendziwiat. Hiltrud, Muhle. Annika, Rademacher. Andreas, van Baalen. Sarah, von Spiczak. Ulrich, Stephani. Zaid, Afawi. Amos, D, Korczyn. Moien, Kanaan. Christina, Canavati. Gerhard, Kurlemann. Karen, Müller-Schlüter. Gerhard, Kluger. Martin, Häusler. Ilan, Blatt. Johannes, R, Lemke. Ilona, Krey. Yvonne, G, Weber. Stefan, Wolking. Felicitas, Becker. Christian, Hengsbach. Sarah, Rau. Ana, F, Maisch. Bernhard, J, Steinhoff. Andreas, Schulze-Bonhage. Susanne, Schubert-Bast. Herbert, Schreiber. Ingo, Borggräfe. Christoph, J, Schankin. Thomas, Mayer. Rudolf, Korinthenberg. Knut, Brockmann. Gerhard, Kurlemann. Dieter, Dennig. Rene, Madeleyn. Reetta, Kälviäinen. Pia, Auvinen. Anni, Saarela. Tarja, Linnankivi. Anna-Elina, Lehesjoki. Mark, I, Rees. Seo-Kyung, Chung. William, O, Pickrell. Robert, Powell. Natascha, Schneider. Simona, Balestrini. Sara, Zagaglia. Vera, Braatz. Michael, R, Johnson. Pauls, Auce. Graeme, J, Sills. Larry, W, Baum. Pak, C, Sham. Stacey, S, Cherny. Colin, H T, Lui. Nina, Barišić. Norman, Delanty. Colin, P, Doherty. Arif, Shukralla. Mark, McCormack. Hany, El-Naggar. Laura, Canafoglia. Silvana, Franceschetti. Barbara, Castellotti. Tiziana, Granata. Federico, Zara. Michele, Iacomino. Francesca, Madia. Maria, Stella, Vari. Maria, Margherita, Mancardi. Vincenzo, Salpietro. Francesca, Bisulli. Paolo, Tinuper. Laura, Licchetta. Tommaso, Pippucci. Carlotta, Stipa. Raffaella, Minardi. Antonio, Gambardella. Angelo, Labate. Grazia, Annesi. Lorella, Manna. Monica, Gagliardi. Elena, Parrini. Davide, Mei. Annalisa, Vetro. Claudia, Bianchini. Martino, Montomoli. Viola, Doccini. Carla, Marini. Toshimitsu, Suzuki. Yushi, Inoue. Kazuhiro, Yamakawa. Birute, Tumiene. Lynette, G, Sadleir. Chontelle, King. Emily, Mountier. Hande, S, Caglayan. Mutluay, Arslan. Zuhal, Yapıcı. Uluc, Yis. Pınar, Topaloglu. Bulent, Kara. Dilsad, Turkdogan. Aslı, Gundogdu-Eken. Nerses, Bebek. Sibel, Uğur-İşeri. Betül, Baykan. Barış, Salman. Garen, Haryanyan. Emrah, Yücesan. Yeşim, Kesim. Çiğdem, Özkara. Annapurna, Poduri. Beth, R, Shiedley. Catherine, Shain. Russell, J, Buono. Thomas, N, Ferraro. Michael, R, Sperling. Warren, Lo. Michael, Privitera. Jacqueline, A, French. Steven, Schachter. Ruben, I, Kuzniecky. Orrin, Devinsky. Manu, Hegde. Pouya, Khankhanian. Katherine, L, Helbig. Colin, A, Ellis. Gianfranco, Spalletta. Fabrizio, Piras. Federica, Piras. Tommaso, Gili. Valentina, Ciullo. Andreas, Reif. Andrew, McQuillin. Nick, Bass. Andrew, McIntosh. Douglas, Blackwood. Mandy, Johnstone. Aarno, Palotie. Michele, T, Pato. Carlos, N, Pato. Evelyn, J, Bromet. Celia, Barreto, Carvalho. Eric, D, Achtyes. Maria, Helena, Azevedo. Roman, Kotov. Douglas, S, Lehrer. Dolores, Malaspina. Stephen, R, Marder. Helena, Medeiros. Christopher, P, Morley. Diana, O, Perkins. Janet, L, Sobell. Peter, F, Buckley. Fabio, Macciardi. Mark, H, Rapaport. James, A, Knowles. Genomic Psychiatry Cohort (GPC). Ayman, H, Fanous. Steven, A, McCarroll. Namrata, Gupta. Stacey, B, Gabriel. Mark, J, Daly. Eric, S, Lander. Daniel, H, Lowenstein. David, B, Goldstein. Holger, Lerche. Samuel, F, Berkovic. Benjamin, M, Neale. See the Appendix for the affiliations of the Epi25 Collaborative members.

## Data availability

The data/analyses presented in the current publication are based on the use of study data from the Epi25 Collaborative (http://epi-25.org/), available with controlled access through dbGaP (https://ncbi.nlm.nih.gov/gap/) and AnVIL project (https://anvilproject.org/data) with the accession number phs001489, and control exome data from dbGAP that are accessible with appropriate permissions under accession numbers phs000473 (Swedish Schizophrenia Study), phs001000 (Leicester UK Heart Study), phs000806 (Ottawa Hear Study), and phs000814 (Italian Atherosclerosis, Thrombosis, and Vascular Study). Supplemental data supporting the analyses presented are available on Mendeley Data (https://doi.org/10.17632/nmmz4wjvxk.1).

## Declaration of Competing Interest

M. Koko reports grants from DAAD, during the conduct of the study; Dr. R. Krause reports grants from FNR, during the conduct of the study; Dr. med. habil. T. Sander reports grants from DFG, during the conduct of the study; Dr. D. R. Bobbili has nothing to disclose; Prof. Dr. M. Nothnagel reports grants from DFG, during the conduct of the study; Dr. P. May reports grants from FNR, during the conduct of the study; Prof. Dr. med. H. Lerche reports grants from DFG, during the conduct of the study.

## Appendix Supplementary materials

Appendix: Supplementary materials including supplemental methods, tables S1-S6, figures S1-S20, and the affiliations of the Epi25 Collaborative members

Image, application 2

## Footnotes

Supplementary material associated with this article can be found in the online version at doi:10.1016/j.ebiom.2021.103588.
