# Supplementary material for: Distinct gene-set burden patterns underlie common generalized and focal epilepsies
Source: eBioMedicine. 2021 Sep 24;72:103588. doi: 10.1016/j.ebiom.2021.103588 (PMC8479647; doi:10.1016/j.ebiom.2021.103588)
Supplement: Supplementary file 2 [file mmc2.docx]

Caption for supplementary material: This online appendix includes the supplemental methods, tables S1-S6, figures S1-S20, and the affiliations of the Epi25 Collaborative members
